# On Some Unrecognised Forms of Mental Disorder

**Published:** 1856-01-01

**Authors:** Forbes Winslow


(*To be continued.*)

---

### Art. VI.—ON SOME UNRECOGNISED FORMS OF MENTAL DISORDER.

BY FORBES WINSLOW, M.D.

In the ordinary practice of medicine we occasionally meet with cases of disease which are at variance with our past experience and *à priori* notions, set at defiance our preconceived views of morbid physical phenomena, resist every attempt to embody them within the *nosological* chart, and which repudiate all reduction to any of the acknowledged orthodox pathological standards or tests. These affections are anomalous or *pseudo* in their character, are, with difficulty defined, not easily diagnosed, occasionally escape observation, and often resist, too successfully, the operation of the best directed remedial measures. If, among the diseases more particularly implicating the ordinary organic functions of life, we witness these pseudo or eccentric deviations from the recognised pathological character, *à fortiori*, are we not justified in anticipating that in the subtle, complicated, varied, and often obscure affections of the cerebral matter, deranging the operations of mind, we should have brought within the sphere of our observation extraordinary, anomalous, and eccentric deviations from certain pre-determined, morbid, cerebral, and psychological conditions? It is the purport of this essay to illustrate some of these spurious morbid mental states. It is not my intention to discuss that *vexata quæstio*, what constitutes insanity, or to lay down rules by which we may successfully trace, in every case, the line of demarcation between the sane and insane condition, passion and insanity, eccentricity and mental derangement.

With the view of avoiding this discussion, I have preferred confining my remarks to those unrecognised forms of what may be properly termed *mental disorders*. I presume it to be a generally admitted axiom that the mind may be *disordered* without being *insane*, using this phrase in its strictly legal acceptation. These conditions of morbid thought may be considered by some as only *degrees* of *insanity;* but I would suggest that this term be restricted to those mental affections accompanied by positive aberration or derangement of idea, associated with loss of controlling power, clearly justifying the exercise of moral restraint; and to those morbid conditions of the intellect which sanction an appeal to the protective influence of the law. In other words, I would confine my remarks to those cases in which the mind may be said to be *pathologically* disordered but not *legally* insane. Have we in practice sufficiently appreciated this distinction? Fearful of committing ourselves to an opinion that might authorize an interference with the free agency of the subject, and justify the use of legal restraint, have we not exhibited an indisposition to admit the existence of positive mental disorder, even in cases where it has been obviously and painfully apparent? This excessive caution—originating in motives that do honour to human nature—has often, I fear, been productive of serious, fatal, and irremediable mischief.

The subject under consideration is one, I readily admit, of extreme delicacy, but one, nevertheless, to my humble conception, of incalculable importance to all sections of the community. It is beset with difficulties and surrounded by dangers. In the hands of the inexperienced, the ignorant, the indiscreet, and the wilfully designing, the facts that I have to record, and principles which I purpose to enunciate, might be productive of much mischief; but, I ask, ought any apprehensions of this kind to deter me from entering upon this important inquiry? The subject of latent and unrecognised morbid mind is yet in its infancy. It may be said to occupy, at present, untrodden and almost untouched ground. What a vast field is here presented to the truth seeking and philosophical observer, who, to a practical knowledge of the world and human character, adds an acquaintance with the higher departments of mental philosophy and a knowledge of cerebral pathology. How much of the bitterness, misery, and wretchedness so often witnessed in the bosom of families arises from concealed and undetected mental alienation! How often do we witness ruin, beggary, disgrace, and death result from such unrecognised morbid mental conditions! It is the canker worm gnawing at the vitals, and undermining the happiness of many a domestic hearth. Can nothing be done to arrest the fearful progress of the moral avalanche, or arrest the course of the rapid current that is hurling so many to ruin and destruction?

This type of morbid mental disorder exists to a frightful extent in real life. It is unhappily on the increase, and it therefore behoves the profession, as guardians of the public health, as medical philosophers engaged in the loftiest and most ennobling of human inquiries, as practical physicians called upon to unravel the mysterious and complicated phenomena of disease, and to administer relief to human suffering, to fearlessly grapple with an evil which is sapping the happiness of families, and to exert their utmost ability to disseminate sound principles of pathology upon a matter so intimately associated and so closely interwoven with the social well-being of the human race. These unrecognised morbid conditions most frequently implicate the affections, propensities, appetites, and moral sense. In many instances it is difficult to distinguish between normal or healthy mental irregularities of thought, passion, appetite, and those deviations from natural conditions of the intellect, both in its intellectual and moral manifestations, clearly bringing those so affected within the legitimate domain of pathology. Are there any unfailing diagnostic symptoms by means of which we may detect these pseudo forms of mental disorder with sufficient exactness, precision, and distinctness to justify the conclusion that they result from a deviation from the normal cerebral condition? This question it will be my duty to consider. The affections of which I speak are necessarily obscure, and, unlike the ordinary cases of mental aberration of every day occurrence, they frequently manifest themselves in either an exalted, depressed, or vitiated state of the moral sense. The disorder frequently assumes the character of a mere exaggeration of some single predominant passion, appetite, or emotion, and so often resembles, in its prominent features, the natural and healthy actions of thought, either in excess of development or irregular in its operations, that the practised eye of the experienced physician can alone safely pronounce the state to be one of disease. I do not refer to mere ordinary instances of eccentricity, to certain idiosyncrasies of thought and feeling, or to cases in which the mind appears to be absorbed by some one idea, which exercises an influence over the conduct and thoughts quite disproportionate to its intrinsic value. Neither do I advert to examples of natural irritability, violence or passion, coarseness and brutality, vicious inclinations, criminal propensities, excessive caprice, or extravagance of conduct, for these conditions of mind may, alas! be the natural and healthy operations of the intellect. These strange phases of the understanding—these *bizarreries* of character—these vagaries of the intellect—these singularities, irregularities, and oddities of conduct, common to so many who mix in every day life, and who pass current in society, present to the philosophical psychologist many points for grave contemplation and even suspicion; but such natural and normal, although eccentric states of the intellect, do not legitimately come within the province of the practical physician unless they can be clearly demonstrated to be *morbid results*—to be positive and clearly established deviations from cerebral and mental health. It has been well observed by Dr. Coombe that a brusque, rough manner, which is natural to one person, indicates nothing but mental health in him, but if another individual, who has always been remarkable for a deferential deportment and habitual politeness, lays these qualities aside, and, without provocation or other adequate cause, assumes the unpolished forwardness of the former, we may justly infer that his mind is either already deranged or on the point of becoming so; or if a person who has been noted all his life for prudence, steadiness, regularity, and sobriety, suddenly becomes, without any adequate change in his external situation, rash, unsettled, and dissipated in his habits, or *vice versâ*, every one recognises at once in these changes, accompanied as they are by certain bodily symptoms, evidences of the presence of disease affecting the mind through the instrumentality of its organs. It is not therefore the abstract feeling or act that constitutes positive proof of the existence of mental derangement, but a departure from, or an exaggeration of, the natural and healthy character, temper, habits of the person so affected.

These forms of unrecognised mental disorder are not always accompanied by any well marked disturbance of the bodily health demanding medical attention, or any obvious departure from a normal state of thought and conduct such as to justify legal interference; neither do these affections always incapacitate the party from engaging in the ordinary business of life. There may be no appreciable morbid alienation of affection. The wit continues to dazzle, and the repartee has lost none of its brilliancy. The fancy retains its playfulness, the memory its power, and the conversation its perfect coherence and rationality. The afflicted person mixes as usual in society, sits at the head of his own table, entertains his guests, goes to the stock-exchange, to his counting-house or his bank, engages actively in his professional duties, without exhibiting evidence, very conclusive to others, of his actual morbid condition. The mental change may have progressed insidiously and stealthily, having slowly and almost imperceptibly effected important molecular modifications in the delicate vesicular nervous neurine of the brain, ultimately resulting in some aberration of the ideas, or alteration of the affections, propensities, and habits.

The party may be an unrecognised monomaniac, and acting under the terribly crushing and despotic influence of one predominant morbid idea, he bringing destruction upon his once happy home and family. His feeling may be perverted and affections alienated; thus engendering much concealed misery within the sacred circle of domestic life. His conduct may be brutal to those who have the strongest claims upon his love, kindness, and forbearance, and yet his mental malady be undetected. He may recklessly, and in opposition to the best counsels and most pathetic appeals, squander a fortune, which has been accumulated after many years of active industry and anxious toil. *He may become vicious and brutal—a tyrant, a criminal, a drunkard, a suicide, and a spendthrift, as the result of an undoubtedly morbid state of the brain and mind, and yet pass unobserved through life as a sane, rational, and healthy man.*

We witness in actual practice all the delicate shades and gradations of such unrecognised and neglected mental alienation. It often occurs that whilst those so affected are able to perform with praiseworthy propriety and with scrupulous probity and singular exactness, most of the important duties of life, they manifest extraordinary and unreasonable antipathies, dislikes, and suspicions against their dearest relations and kindest friends. So cleverly and successfully is this mask of sanity and mental health sometimes worn; so effectually is all suspicion disarmed, that mental disorder of a dangerous character has been known for years to progress without exciting the slightest notion of its presence, until some sad and terrible catastrophe has painfully awakened attention to its existence. Persons suffering from latent insanity often affect singularity of dress, gait, conversation, and phraseology. The most trifling circumstances rouse their excitability,—they are martyrs to ungovernable paroxysms of passion, are roused to a state of demoniacal furor by insignificant causes, and occasionally lose all sense of delicacy of feeling and sentiment, refinement of manners and conversation. Such manifestations of undetected mental disorder are often seen associated with intellectual and moral qualities of the highest order. Neither rank nor station is free from these sad mental infirmities. Occasionally the malady shows itself in an overbearing disposition. Persons so unhappily disordered browbeat and bully those over whom they have the power of exercising a little short-lived authority, and, forgetting what is due to station, intelligence, reputation, and character, they become within their circumscribed sphere petty tyrants, aping the manners of an Eastern despot. They are impulsive in their thoughts, are often obstinately and pertinaciously rivetted to the most absurd and outrageous opinions, are dogmatic in conversation, are litigious, exhibit a controversial spirit, and oppose every endeavour to bring them within the domain of common sense and correct principles of reasoning. Persons, who were distinguished for their sweetness of disposition, unvarying urbanity, strict regard for truth, diffidence of character, evenness of temper, and all those self-denying qualities which adorn and beautify the human character, exhibit, in this type of disordered intellect, states of morbid mind the very reverse of those natural to them when in health. The even-tempered man becomes querulous and irascible; the generous and open-hearted become cunning and selfish; the timid man assumes an unnatural boldness and forwardness. All delicacy and decency of thought is occasionally banished from the mind, so effectually does the spiritual principle in these attacks succumb to the animal instincts.

The naturally gentle, truthful, retiring, and self-denying, become quarrelsome, cunning, and selfish—the diffident bold, and the modest obscene. We frequently observe these pseudo-mental conditions involving only one particular faculty, or seizing hold of one passion or appetite. Occasionally it manifests itself in a want of veracity, or in a disposition to exaggerate, amounting to positive disease. It may show itself in a disordered volition, in morbid imitation, in an inordinate vaulting ambition, an absorbing lust of praise, an insane desire for notoriety, a sudden paralysis of the memory or impairment of the power of attention, with an obliteration from the mind of all the events of the past life. The disorder occasionally manifests itself in a depressed, exalted, or vitiated state of the reproductive function—in morbid views of Christianity, and is often connected with a profound *anæsthesia*

of the moral sense. Many of these sad afflictions are symptomatic of unobserved, and, consequently, neglected cerebral conditions, either originating in the brain itself, or produced by sympathy with morbid affections existing in other tissues in close organic relationship with the great nervous centre.

The majority of these cases will generally be found associated with a constitutional predisposition to insanity and cerebral disease. These morbid conditions are occasionally the sequelæ of febrile attacks, more or less implicating the functions of the brain and nervous system. They often succeed injuries of the head inflicted in early childhood; and modifications of the malady are also, unhappily, seen allied with genius; and, as the biographies of Cowper, Burns, Byron, Johnson, Pope, and Haydon establish, the best, the exalted, and most highly gifted conditions of mind do not escape unscathed. In early childhood this form of mental disturbance may be detected in many cases. To its existence may often be traced the motiveless crimes of the young, as well as much of the unnatural caprice, dulness, stupidity, and wickedness often witnessed in early life. In the majority of instances, the patient is quite ignorant of his condition, and indignantly repudiates the imputation of mental ill health. In some cases, however, the unhappy sufferer is perfectly conscious of his lamentable state, and, feeling a necessity for cerebral relief, eagerly seeks the advice and consolation of his confidential physician. In this stage of mental consciousness, a painful struggle often takes place in the patient's mind relative to the reality of his mental impressions or suggestions. The questions occasionally occurring to the mind are as follow:—Are these ideas consistent with health? is there any basis for such thoughts? am I justified in harbouring feelings of this nature? are they false creations, or notions of a healthy character, arising out of actual circumstances? A battle of this kind, with ideas clearly of a morbid character, I have known to continue for a long period before the intellect has become prostrated, or succumbed to the insane delusion, or suicidal suggestion. This type of case often comes under the notice of those engaged in the treatment of mental maladies.

Hamlet, when he imagined his soundness of mind questioned, exclaims—

> "This is not madness, bring me to the test."

Again: Shakespeare makes Lady Constance, when accused of insanity, in consequence of her intense manifestations of grief, declare

> "I am not mad."

She then proceeds to describe to her accuser her reasons for repudiating the imputation of insanity;—

"I am not mad; this hair I tear is mine;
My name is Constance;
Young Arthur is my son, and *he* is lost.
I am not mad;—I would to Heaven I were;
For then, 'tis like I should forget myself.
O, if I could, what grief should I forget!"

Then, in the bitterness of wild despair, she begs the Cardinal to "preach some philosophy to make her mad," for she exclaims—

"Being not mad, but sensible of grief,
My reasonable part produces reason;
If I were mad, I should forget my son,
Or madly think a babe in clouts were he."

Again: overpowered by the terrible consciousness of her sad condition, she thus repeats her declaration of sanity—

"I am not mad; too well, too well I feel
The different plague of each calamity."

This condition of mind is closely allied to positive insanity. In this stage of consciousness the disorder easily yields to medical treatment.

It is unnecessary for me to direct attention to the frightful amount of unrecognised and untreated cases of mental depression associated with an irresistible suicidal propensity which has prevailed, within the last twelve or eighteen months. The daily channels of communication convey to us this sad intelligence in language that does not admit of misconstruction. The melancholy history of one case recorded is but a faithful record of hundreds of others that are occurring within the range of our own vision. If the evidence generally adduced before the coroner is to be credited, in nearly every case of suicide, cerebral disorder has exhibited itself, and the mind has been clearly and palpably deranged. In many cases, the mental alienation has clearly existed for weeks, and occasionally for months, without giving rise to the suspicion of the presence of any dangerous degree of brain disturbance likely to lead to an overt act of suicide. There are few morbid mental conditions so fatal in their results, as these apparently trifling, evanescent, and occasionally fugitive attacks of mental depression. They almost invariably, in certain temperaments, are associated with suicidal impulse. I am never consulted in a case of this character without fully impressing upon the relatives and friends the importance of careful vigilance. These slight ruffles upon the surface, these attacks of mental despondency, these paroxysms of morbid *ennui*, accompanied as they generally are with intense weariness of life, a desire for seclusion, love of solitude, and a want of interest in the ordinary affairs of life, are fraught with fatal mischief. How much of this character of disordered mind not only escapes observation, but is subjected to no kind of medical and moral treatment. Occasionally it may happen (but how rare is the occurrence), that the unhappy suicide may have exhibited no appreciable symptoms of mental derangement; but even in these cases we should be cautious in concluding that sanity existed at the time of the suicide. It often happens that a person is impelled to self-destruction by the overpowering and crushing influence of some latent and concealed delusion, that has for weeks, and perhaps for months, been sitting like an incubus upon the imagination. Patients often confess that they have been under the influence of monomaniacal ideas and concealed hallucinations for months without their existence being suspected even by their most intimate associates. "For six months," writes a patient, "I have never had the idea of suicide, night or day, out of my mind. Wherever I go, an unseen dæmon pursues me, impelling me to self-destruction. My wife, my friends, my children, observe my listlessness and my despondency, but they know nothing of the worm that is gnawing within." Is this not a type of case more generally prevalent than we imagine? May we not say of this unhappy man, with a mind tortured and driven to despair by a terrible, overpowering, and concealed delusion, urging him on to suicide, as the only escape and relief from the acuteness of his misery,

"HE hears a voice WE cannot hear,
  Which says, he must not stay,
HE sees a hand WE cannot see,
  Which beckons him away"?

